# Role of Bcl-2 -938 C>A polymorphism in susceptibility and prognosis of cancer: a meta-analysis

**DOI:** 10.1038/srep07241

**Published:** 2014-11-28

**Authors:** Xiao Zhang, Wenhao Weng, Wen Xu, Yulan Wang, Wenjun Yu, Xun Tang, Lifang Ma, Qiuhui Pan, Jiayi Wang, Fenyong Sun

**Affiliations:** 1 Department of Clinical laboratory medicine, Shanghai Tenth People's Hospital of Tongji University, Shanghai, China, 200072; 2 Department of Clinical laboratory medicine, Zhongshan Hospital, Fudan University, Shanghai, China, 200032; 3 Department of Central Laboratory, Shanghai Tenth People's Hospital of Tongji University, Shanghai, China, 200072

## Abstract

The association between B-cell lymphoma 2 (*Bcl-2*) polymorphism and cancer is under debate and remains elusive. This meta-analysis was performed to evaluate the relationships of *Bcl-2* -938 C>A polymorphism (rs2279115) with susceptibility and prognosis of cancer. Odds ratios (ORs) were used to measure the association between *Bcl-2* polymorphisms and cancer risk. Hazard ratios (HRs) were used to measure the association between *Bcl-2* polymorphisms and cancer prognosis. On the basis of 26 studies about *Bcl-2* -938C>A polymorphism and cancer, we found *Bcl-2* -938 C>A polymorphism was significantly associated with increased cancer risk in dominant model (OR = 1.12, 95%CI: 1.00–1.25, P = 0.04), recessive model (OR = 1.38, 95%CI: 1.11–1.71, P = 0.004), allelic model (OR = 1.15, 95%CI: 1.04–1.28, P = 0.007) and homozygote comparison(OR = 1.44, 95%CI: 1.11–1.87, P = 0.006). Furthermore, *Bcl-2* -938 C>A polymorphism was significantly associated with increased cancer risk in Asians but not in Caucasians. Moreover, *Bcl-2* -938 C>A polymorphism was not significantly associated with the prognosis of cancer (AA vs CA: OR = 0.99, 95%CI: 0.77–1.27, P = 0.93; AA vs CC: OR = 0.92, 95%CI: 0.65–1.30, P = 0.63; AC vs CC: OR = 0.94, 95%CI: 0.80–1.11, P = 0.48; CC vs AA+CA: OR = 1.21, 95%CI: 0.69–2.13, P = 0.50; AA vs CC+CA: OR = 0.99, 95%CI: 0.48–2.04, P = 0.97). Studies with larger samples and gene-environment interactions are needed to validate our findings.

Apoptosis is a highly programmed cell death, and it can be achieved by two major pathways: death-receptor pathway and mitochondrial pathway^1^. The Bcl-2 family proteins play an important role in the regulation of the mitochondrial pathway of apoptosis through controlling the outer mitochondrial membrane integrity^2^. Bcl-2 family contains more than 20 anti-apoptotic and pro-apoptotic members such as Bcl-2, Bax, Bad and Bak^3^. Bcl-2 is highly expressed at the onset of many cancers^4^. High expression of Bcl-2 has been reported in solid-tumors like prostate cancer^5^ and non-small cell lung cancer^6^. In blood cancers like chronic lymphocytic leukemia^7^ and diffuse large B-cell lymphoma^8^, high expression of Bcl-2 was also reported.

*Bcl-2* (B-cell leukemia/lymphoma 2) gene, located at 18q21.3^9^, which is firstly identified as an anti-apoptotic regulatory protein, and served as an inhibitor of proliferation^10^. *Bcl-2* consists of two promoters which have different functions named P1 and P2^11^. Previous studies have identified a novel single-nucleotide polymorphism (-938 C>A) in P2 promoter of the *Bcl-2* gene^12^. *Bcl-2* -938C>A polymorphism is a crucial factor of cell cycle control and cell survival^13^. Wedemeyer et al. reported that *Bcl-2* -938 CC genotype is at high risk for aseptic loosening^14^. Zhang et al. reported that *Bcl-2* -938C>A polymorphism may be relevant to the clinical symptoms of major depressive disorder^15^. Recently, several studies have reported that *Bcl-2* -938C>A promoter polymorphism is associated with susceptibility and prognosis of cancer^16^,^17^,^18^,^19^,^20^,^21^,^22^,^23^,^24^,^25^,^26^,^27^,^28^,^29^,^30^,^31^,^32^,^33^,^34^,^35^,^36^,^37^,^38^,^39^.

The aim of the present study was to investigate whether *Bcl-2* -938C>A polymorphism can influence the susceptibility of cancer and to evaluate the prognostic significance of *Bcl-2* -938C>A polymorphism in cancer.

## Methods

### Literature search

The PRISMA statement (Supplementary Checklist S1) were followed in our meta-analysis. PubMed, EMBASE, OVID, Cochrane Library and Web of Science databases were searched from database inception to August 2014 without language restriction. The search strategy was “Bcl-2 OR Bcl2 OR B-cell lymphoma-2” AND “polymorphism or variant or mutation or genotype”. The review articles and reference lists of retrieved articles were read manually to complete our research. The database search was performed independently by X. Zhang and J. Wang and the disagreements were resolved through consensus by all of the authors.

### Selection criteria

If the following inclusion were satisfied, studies would be included in our meta-analysis: 1)case-control studies focused on association between the *Bcl-2* promoter polymorphism (-938 C>A) and susceptibility or prognostic significance in cancer. 2) More than 30 patients and controls were enrolled in studies.3) Studies provided sufficient data to estimate the odds ratio (OR) or hazard ratio (HR) and 95% confidence intervals (CI) according to *Bcl-2* promoter polymorphism(-938 C>A). 4) When study patients overlapped with patients in other included studies, we selected the first study published. The two researchers (J. Wang and X. Zhang) read the titles and abstracts independently and excluded the uncorrelated studies; then the full-texts were examined by our review team. The studies would be selected according to the inclusion criteria.

### Data Abstraction

The following information in studies investigating the association between *Bcl-2* polymorphism and cancer risk was extracted by two independent researchers: authors, year of publication, country, tumor type, number of cases and controls analyzed, mean value of age, source of controls (hospital-based controls or population-based controls) and genotyping method. As for studies investigating the association between *Bcl-2* polymorphism and prognostic value in cancer, two researchers independently extracted the following information from the article: authors, year of publication, country, tumor type, number of patients analyzed, distribution of age and gender, genotyping method, HR estimation and median follow-up date. If univariate and multivariate analysis were both reported, we selected the multivariate analysis. Because the multivariate analysis has taken into consideration the confounding factor and is more accurate. If insufficient data (missing data, inconsistencies, or any other uncertainties) were reported in the article, we tried our best to ask the first and corresponding authors for necessary information by telephone or E-mail.

### Statistical analysis

As for studies investigating the association between *Bcl-2* -938C>A polymorphism and cancer susceptibility, odds ratios (ORs) and corresponding 95% confidence intervals (CIs) were combined to measure the association between *Bcl-2* promoter polymorphisms and susceptibility of cancer. Hardy-Weinberg equilibrium (HWE) for each study was determined by Chi square test. The pooled ORs were calculated for the dominant model (WM + MM vs WW), recessive model (MM vs WM + WW), homozygote comparison (MM vs WW), heterozygote comparison (WM vs WW) and allelic model (mutation [M] allele versus [vs] wild [W] allele), respectively. As for studies evaluating the prognostic significance of *Bcl-2* -938C>A polymorphism in cancer, hazard ratios (HRs) and corresponding 95% confidence intervals (CIs) were combined to measure the effective value of *Bcl-2* -938 C>A polymorphism on prognosis. If the study didn't report the HRs, the Engauge Digitizer version 4.1 was used to read the kaplane-Meier curves to estimate the HRs and the 95% CIs. In order to reduce reading variability, three independent investigators (J. Wang, W. Weng and X. Zhang) read the curves. P values<0.05 indicated statistical significance. Statistical heterogeneity among the studies was evaluated using the Q test and I^2^ test. When heterogeneity among the studies was observed, the pooled OR/HR was calculated by random-effects models. Sensitivity analyses were performed to identify the potential influence of the individual data set to the pooled OR/HR. These analyses were performed by Review Manager Version 5.1 software (http://ims.cochrane.org/revman). The Begg's and Egger's test was performed by R (http://cran.r-project.org/bin/windows/base). We applied re-sampling statistic and 1000 re-sampling groups were generated using the bootstrap re-sampling procedure^40^,^41^. The re-sampling program was in Supplementary excel file 1, and one of the result was displayed in Supplementary excel file 2. In our re-sampling program (Supplementary excel file 1), the types of each 11805 samples were showed. No.1 was for cancer patient and CC genotype; 2 was for cancer patient and CA genotype; 3 was for cancer patient and AA genotype; 4 was for healthy control and CC genotype; 5 was for healthy control and CA genotype; 6 was for healthy control and AA genotype. In one re-sampling group, there were 11805 samples generated by bootstrap re-sampling procedure and the ORs were calculated under five genetic models. 1000 re-sampling groups were generated to get robust and replicable results. Overall ORs were calculated containing all samples under five genetic models. Pressing F9 was able to re-sample. In one of the re-sampling results (Supplementary excel file 2), the distributions of ORs in five genetic models were analyzed, and the overall ORs containing all samples were calculated under five genetic models.

## Results

### Characteristics of identified studies

**Figure 1:**
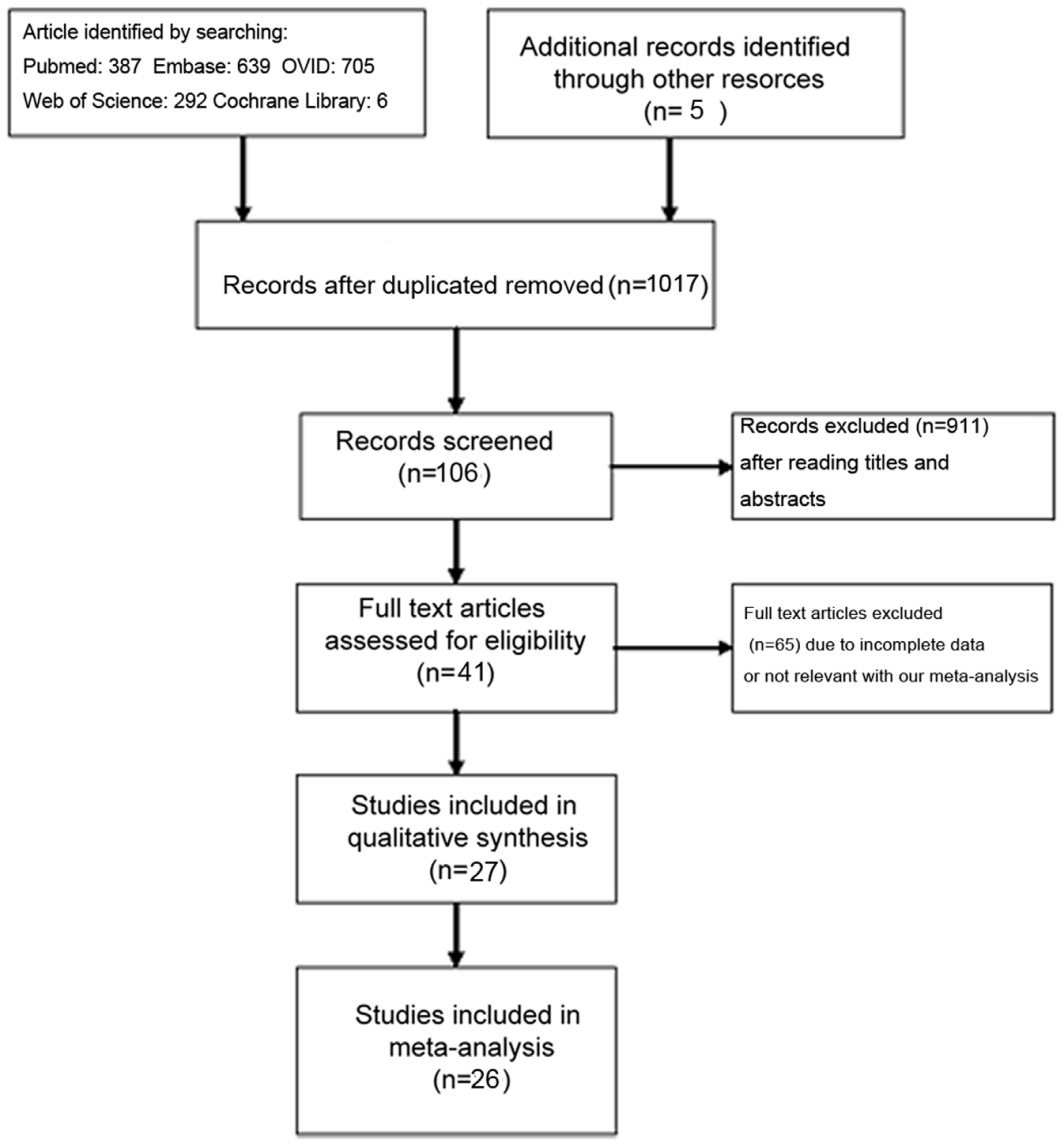
Flow diagram summarizing the selection of eligible studies.

Following an initial search, 387 studies were searched in PubMed, 639 studies were searched in EMBASE, 705 studies were searched in OVID, 6 studies were searched in Cochrane Library, 292 studies were searched in Web of Science and 5 additional studies in review article were added to make our search comprehensive. 1017 published studies were identified after duplicated records removed. After excluding unrelated studies by reading titles, abstracts and the full-text, trying our best to communicate with the first and corresponding author to get the necessary data, 26 studies were included in our meta-analysis ultimately. Twelve studies evaluating *Bcl-2* -938 C>A polymorphism in cancer risk^28^,^29^,^30^,^31^,^32^,^33^,^34^,^35^,^36^,^37^,^38^,^39^ and fourteen studies evaluating the prognostic value of *Bcl-2* -938 C>A polymorphism in cancer^16^,^17^,^18^,^19^,^20^,^21^,^22^,^23^,^24^,^25^,^26^,^27^,^30^,^38^ were included in our meta-analysis. The detailed selection process was displayed in Figure 1. As for studies investigating the association between *Bcl-2* -938C>A polymorphism and cancer susceptibility, studies were published between 2007 and 2014. There were 5515 cases and 6290 controls included in our meta-analysis. Studies were carried out in China, Germany and USA. Two studies assessed prostate cancer^31^,^36^, and one each for glioma^29^, breast cancer^35^, thyroid carcinoma^33^, non-Hodgkin lymphoma^28^, squamous cell carcinoma of the head and neck^39^, esophageal cancer^34^, lung cancer^30^, extrahepatic cholangiocarcinoma^37^, endometrial cancer^32^ and chronic lymphocytic leukemia^38^. As for studies evaluating the prognostic significance of *Bcl-2* -938C>A polymorphism in cancer, studies were published between 2007 and 2013 and carried out in Japan, Korea, Sweden, China and Germany. Five studies assessed leukemia^17^,^21^,^25^,^26^,^38^, three studies assessed lung cancer^16^,^18^,^30^ and one each for renal cancer^23^, prostate cancer^20^, breast cancer^27^, squamous cell carcinoma^22^, glioblastoma and ovarian cancer^19^. The main characteristics of all the included studies is shown in Supplementary Table S1 and Supplementary Table S2.

### *Bcl-2* -938C>A polymorphism and cancer susceptibility

**Figure 2:**
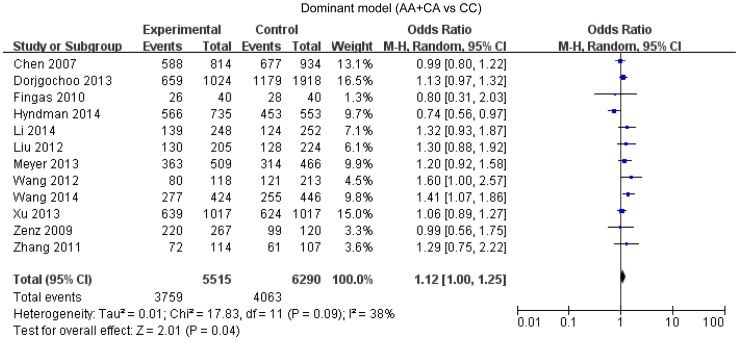
Forest plot of *Bcl-2* -938 C>A polymorphism and cancer risk in dominant model.

**Figure 3:**
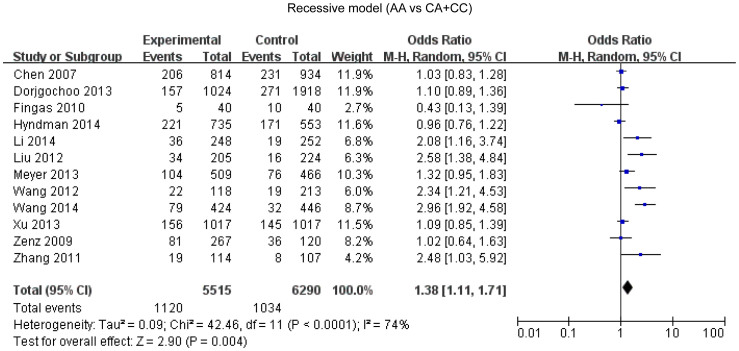
Forest plot of *Bcl-2* -938 C>A polymorphism and cancer risk in recessive model.

**Figure 4:**
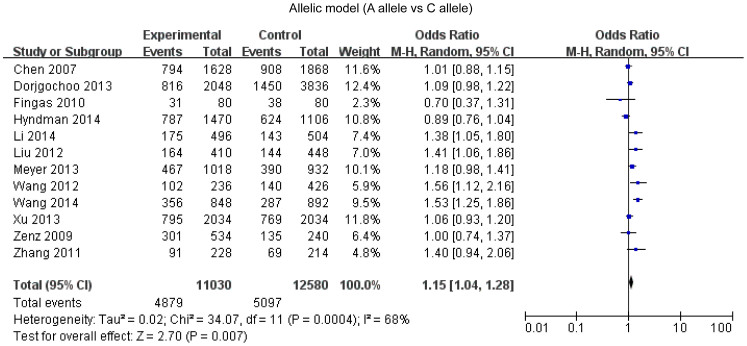
Forest plot of *Bcl-2* -938 C>A polymorphism and cancer risk in allelic model.

**Figure 5:**
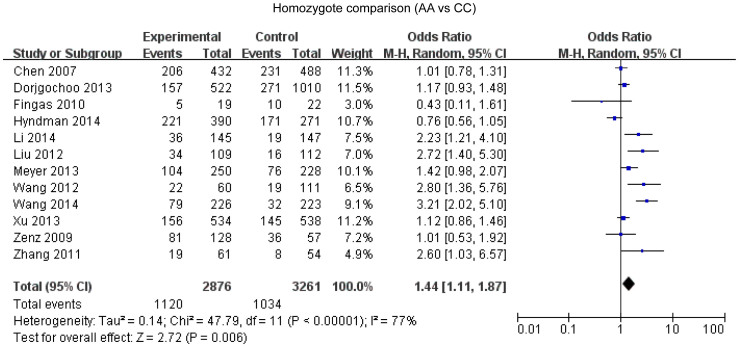
Forest plot of *Bcl-2* -938 C>A polymorphism and cancer risk in homozygote comparison.

Overall, twelve studies enrolling 5515 cases and 6290 controls were included in our meta-analysis. A statistical significant association between *Bcl-2* -938 C>A polymorphism and cancer susceptibility was found under the dominant model (OR = 1.12, 95%CI: 1.00-1.25, P = 0.04) (Fig. 2), recessive model (OR = 1.38, 95%CI: 1.11–1.71, P = 0.004) (Fig. 3), allelic model (OR = 1.15, 95%CI: 1.04–1.28, P = 0.007) (Fig. 4) and homozygote comparison(OR = 1.44, 95%CI: 1.11–1.87, P = 0.006) (Fig. 5). And no significant association was found under the heterozygote comparison (OR = 1.05, 95%CI: 0.97–1.14, P = 0.22) (Supplementary Figure S1). *Bcl-2* -938 C>A polymorphism was significantly associated with increased cancer risk in Asians under five genetic models (dominant model: OR = 1.19, 95%CI: 1.08–1.31, P = 0.0005; recessive model: OR = 1.83, 95%CI: 1.28–2.62, P = 0.0009; allelic model: OR = 1.28, 95%CI:1.12–1.47, P = 0.0003; homozygote comparison: OR = 1.96, 95%CI: 1.35–2.85, p = 0.006; heterozygote comparison: OR = 1.11, 95%CI: 1.11–1.23, P = 0.04). However, *Bcl-2* -938 C>A polymorphism was not significantly associated with cancer risk in Caucasians (dominant model: OR = 0.96, 95%CI: 0.79–1.16,P = 0.65; recessive model: OR = 1.04, 95%CI: 0.89–1.21, P = 0.82; allelic model: OR = 1.00, 95%CI: 0.89–1.12, P = 0.97; homozygote comparison: OR = 0.98, 95%CI:0.75–1.29, P = 0.91; heterozygote comparison: OR = 0.94, 95%CI:0.80–1.11, P = 0.48). Supplementary Table S3 displays the results of overall and subgroup analysis.

### Sensitivity analysis

Sensitivity analysis was performed by omitting one study at a time and calculating the pooled ORs again. We performed sensitivity analysis in five different genetic models (Supplementary Table S4–S8). When the study performed by Hyndman^36^ was omitted in dominant model, *Bcl-2* -938 C>A polymorphism was associated with increased cancer susceptibility (OR = 1.15, 95%CI: 1.06–1.25, P = 0.001), and the heterogeneity was obviously reduced.

### Publication bias

Both Begg's funnel plot and Egger's test were performed to evaluate the publication bias of the studies. Table S9, Supplementary Figure S2 and Supplementary Figure S3 showed the detailed results. No publication bias was found under five genetic models according to Begg's funnel plot and Egger's test.

### Overall analysis

We evaluated the meta-analysis of all cancer samples that was treating them as a cancer group against the control group to evaluate the significance of the odds ratios. *Bcl-2 -*938 C>A polymorphism was significantly associated with increased cancer risk in five genetic models. In dominant model, OR was 1.17, 95%CI: 1.09–1.27, P<0.0001 (Supplementary Figure 4); In recessive model, OR was 1.30, 95%CI: 1.18–1.42, P<0.00001 (Supplementary Figure 5); In homozygote comparison, OR was 1.37, 95%CI: 1.24–1.53, P<0.00001 (Supplementary Figure 6); In heterozygote comparison, OR was 1.10. 95%CI: 1.02–1.20, P = 0.02 (Supplementary Figure 7); In allelic model, OR was 1.16, 95%CI: 1.11–1.23, P<0.00001 (Supplementary Figure 8).

### Re-sampling statistics

In order to obtain robust and replicable results in our meta-analysis, we applied bootstrap re-sampling procedures. Results were displayed in Supplementary excel file 2. In dominant model, odds ratios were mostly distributed between 1.065 and 1.285 in 1000 re-sampling groups. The odds ratio was 1.17 when evaluating 11805000 samples (95% CI: 1.17–1.17, P<0.00001). In recessive model, odds ratios were mostly distributed between 1.095 and 1.365 in 1000 re-sampling groups. The odds ratio was 1.22 when evaluating 11805000 samples (95% CI: 1.22–1.22, P<0.00001). In homozygote comparison, odds ratios were mostly between 1.135 and 1.465. The odds ratio was 1.30 when evaluating 6146976 samples (95%CI: 1.30–1.31, P<0.00001). In heterozygote comparison, odds ratios were mostly distributed between 1.02 and 1.215 in 1000 re-sampling groups. The odds ratio was 1.12 when evaluating 9522955 samples (95% CI: 1.12–1.12, P<0.00001). In allelic model, odds ratios were mostly distributed between 1.065 to 1.215 in 1000 re-sampling groups. The odds ratio was 1.14 when evaluating 11805000 samples (95%CI: 1.14–1.14, P<0.00001).

### *Bcl-2* -938C>A polymorphism and prognostic significance

**Figure 6:**
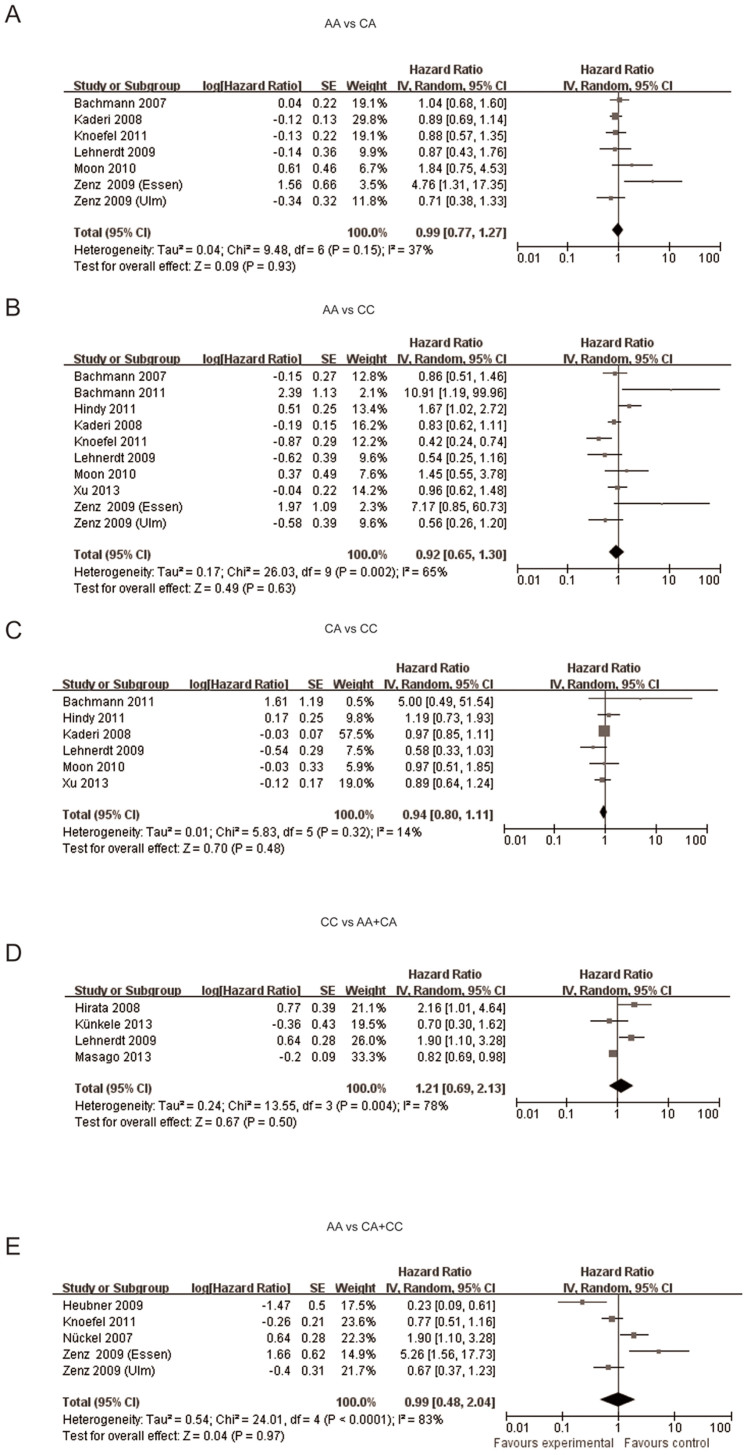
Forest plot of *Bcl-2* -938 C>A polymorphism and cancer prognosis in five genetic models. (A) Forest plot of *Bcl-2* -938 C>A polymorphism and cancer prognosis in AA vs CA; (B) Forest plot of *Bcl-2* -938 C>A polymorphism and cancer prognosis in AA vs CC; (C) Forest plot of *Bcl-2* -938 C>A polymorphism and cancer prognosis in CA vs CC (D) Forest plot of *Bcl-2* -938 C>A polymorphism and cancer prognosis in CC vs AA+CA; (E) Forest plot of *Bcl-2* -938 C>A polymorphism and cancer prognosis in AA vs CA+CC.

Fourteen studies evaluating the prognostic value of *Bcl-2* -938 C>A polymorphism in cancer were included in our meta-analysis. The results of our meta-analysis suggested that the *Bcl-2* -938 C>A polymorphism was not significantly associated with the prognosis of cancer (AA vs CA: OR = 0.99, 95%CI: 0.77–1.27, P = 0.93; AA vs CC: OR = 0.92, 95%CI: 0.65–1.30, P = 0.63; CA vs CC: OR = 0.94, 95%CI: 0.80–1.11, P = 0.48; CC vs AA+CA: OR = 1.21, 95%CI: 0.69–2.13, P = 0.50; AA vs CC+CA: OR = 0.99, 95%CI: 0.48–2.04, P = 0.97) (Figure 6). Sensitivity analysis was performed and the results didn't show any statistical significant difference when any study was omitted (Supplementary Table S10-Table S14). Begg's funnel plot and Egger's test were performed, and no significant publication bias was found (Supplementary Table S15).

## Discussion

Bcl-2 is the founding member of the Bcl-2 family of regulator proteins that regulate cell death. Bcl-2 is specifically considered as an important anti-apoptotic protein and is thus classified as an oncogene^42^. There is increasing evidence that *Bcl-2* gene polymorphism may be associated with cancer susceptibility and prognosis. Recently, polymorphism in *Bcl-2* gene, variant in promoter region -938 C>A (rs2279115), has been reported to be associated with cancer susceptibility and prognosis many times. This might be the first meta-analysis regarding *Bcl-2* polymorphism in cancer susceptibility and prognosis significance.

On the basis of 26 studies about *Bcl-2* -938C>A polymorphism and cancer, we found that *Bcl-2* -938 C>A polymorphism was significantly associated with increased cancer risk in dominant model (OR = 1.12, 95%CI: 1.00–1.25, P = 0.04), recessive model (OR = 1.38, 95%CI: 1.11–1.71, P = 0.004), allelic model (OR = 1.15, 95%CI: 1.04–1.28, P = 0.007) and homozygote comparison(OR = 1.44, 95%CI: 1.11–1.87, P = 0.006). *Bcl-2* -938 C>A polymorphism was significantly associated with increased cancer risk in Asian people (dominant model: OR = 1.19, 95%CI: 1.08–1.31, P = 0.0005; recessive model: OR = 1.83, 95%CI: 1.28–2.62, P = 0.0009; allelic model: OR = 1.28, 95%CI:1.12–1.47, P = 0.0003; homozygote comparison: OR = 1.96, 95%CI: 1.35–2.85, p = 0.006; heterozygote comparison: OR = 1.11, 95%CI: 1.11–1.23, P = 0.04) but not in Caucasian people (dominant model: OR = 0.96, 95%CI: 0.79–1.16,P = 0.65; recessive model: OR = 1.04, 95%CI: 0.89–1.21, P = 0.82; allelic model: OR = 1.00, 95%CI: 0.89–1.12, P = 0.97; homozygote comparison: OR = 0.98, 95%CI:0.75–1.29, P = 0.91; heterozygote comparison: OR = 0.94, 95%CI:0.80–1.11, P = 0.48). Furthermore, *Bcl-2* -938 C>A polymorphism was not significantly associated with the prognosis of cancer (AA vs CA: OR = 0.99, 95%CI: 0.77–1.27, P = 0.93; AA vs CC: OR = 0.92, 95%CI: 0.65–1.30, P = 0.63; AC vs CC: OR = 0.94, 95%CI: 0.80–1.11, P = 0.48; CC vs AA+CA: OR = 1.21, 95%CI: 0.69–2.13, P = 0.50; AA vs CC+CA: OR = 0.99, 95%CI: 0.48–2.04, P = 0.97).

Bcl-2 plays the canonical anti-apoptotic role and has an inhibitory effect on cell-cycle progression. Bcl-2 acts at two different intracellular compartments, the mitochondria and the endoplasmic reticulum^43^. At the mitochondria, Bcl-2 can interact with Bax/Bak via its hydrophobic groove composed of the BH domain 1, 2 and 3, prevent their oligomerization and inhibit Bax/Bak-pore formation. However, small molecules(like BH3 mimetics) can disrupt this interaction, resulting in apoptotic cell death in cancer cells^44^. At the endoplasmic reticulum, Bcl-2 directly binds and inhibits the inositol 1,4,5-trisphosphate receptor (IP3R) via its N-terminal BH4 domain to promote proliferation and increase resistance to apoptosis. If the Bcl-2's inhibitory action on IP3R is reversed, pro-apoptotic Ca^2+^ signaling will be triggered in cancer-B cells^43^. The overexpression of Bcl-2 is seen at the onset of many cancers, like prostate cancer^45^, non-small cell lung cancer^46^, and chronic lymphocytic leukemia^47^. Nowadays, polymorphism in *Bcl-2* gene, variant in promoter region -938 C>A (rs2279115) has been noticed. *Bcl-2* -938C>A polymorphism might become a novel maker in cancer susceptibility and prognosis.

The association between *Bcl-2* -938C>A polymorphism and susceptibility and prognosis in cancer was carefully investigated. However, some limitations might exist in our meta-analysis. Firstly, in the subgroup analysis, there might be insufficient statistical power to check an association. Secondly, although some authors like Xiao-ou Shu^32^ and Martin Heubner^24^ kindly provided necessary data for us, a few authors of studies with incomplete data didn't reply on us. So we couldn't include more studies in our meta-analysis.

In conclusion, this meta-analysis indicates that *Bcl-2* -938C>A polymorphism might be associated with increased cancer risk and this association might exist in Asians but not in Caucasians. Moreover, *Bcl-2* -938C>A polymorphism was not associated the prognostic significance in cancer. Therefore, well-designed prospective studies including the *Bcl-2* -938C>A polymorphism and cancer susceptibility or cancer prognosis with larger sample sizes are needed to validate our findings.

## Supplementary Material

Supplementary Information Supplementary figures

Supplementary Information Supplementary excel file 1

Supplementary Information Supplementary excel file 2
