# Supplementary material for: Role of Bcl-2 -938 C>A polymorphism in susceptibility and prognosis of cancer: a meta-analysis
Source: Sci Rep. 2014 Nov 28;4:7241. doi: 10.1038/srep07241 (PMC5384243; doi:10.1038/srep07241)
Supplement: Supplementary Information — Supplementary figures [file srep07241-s1.pdf]

Role of Bcl-2 -938 C>A polymorphism in susceptibility and prognosis of cancer: a meta-analysis

Xiao Zhang[1], Wenhao Weng[1], Wen Xu[2], Yulan Wang[1], Wenjun Yu[1], Xun Tang[1], Lifang Ma[1], Qiuhui Pan[3], Jiayi Wang[1,*], Fenyong Sun[1,*]

1. Department of Clinical laboratory medicine, Shanghai Tenth People's Hospital of Tongji University, Shanghai, China, 200072;
2. Department of Clinical laboratory medicine, Zhongshan Hospital, Fudan University, Shanghai, China, 200032
3. Department of Central Laboratory, Shanghai Tenth People's Hospital of Tongji University, Shanghai, China, 200072;

Correspondence should be also sent to: *Jiayi Wang, ph.D. E-mail: karajan2@163.com Address: No.301 Middle Yanchang Rd, Shanghai Tenth People's Hospital of Tongji University, Shanghai, China, 200072. Fax: 86-21- 66303643 Tel: 86-21-66300588

Correspondence should be sent to: *Fenyong Sun, M.D., ph.D. E-mail: sunfenyongtongji@126.com, Address: No.301 Middle Yanchang Rd, Shanghai Tenth People's Hospital of Tongji University, Shanghai, China, 200072. Fax: 86-21- 66300588, Tel: 86-21-66300588

Key words: Bcl-2, polymorphism, prognosis, cancer, susceptibility, meta-analysis

Table S1. Baseline characteristics of studies evaluating cancer susceptibility included in the meta-analysis.

Study	Year	Country	Tumor Type	Cases	Controls	Mean Age	Source of controls	Genotyping method	HWE
Hyndman[16]	2011	USA	prostate cancer	735	553	NR	HB	Affymetrix gene chip SNP analysis	0.62
Li [17]	2014	China	glioma	248	252	Case:52.34±14.56 Control:54.22±14.33	HB	PCR-RFLP	0.36
Zhang [18]	2011	China	breast cancer	114	107	NR	PB	PCR-RFLP	0.57
Wang [19]	2012	China	thyroid carcinoma	118	213	NR	HB	PCR-RFLP	0.44
Wang [20]	2014	China	non-Hodgkin lymphoma	424	446	NR	HB	PCR-RFLP	0.26
Chen [21]	2007	USA	squamous cell carcinoma of the head and neck	814	934	NR	HB	PCR-RFLP	0.03
Liu [22]	2012	China	esophageal cancer	205	224	NR	PB	PCR-RFLP	0.27
Xu [23]	2013	China	lung cancer	1017	1017	Case:60.3±10.7 Control:59.7±12.1	PB	TaqMan method using the ABI 7900HT Sequence Detection System	0.97
Fingas [24]	2010	Germany	extrahepatic cholangiocarcinoma	40	40	Case:61.3±12.7	PB	TaqMan method using the ABI 7900HT Sequence Detection System	0.96
Meyer [25]	2013	Germany	prostate cancer	509	466	66.3	HB	TaqMan Universal PCR Master Mix	0.28
Dorjgochoo[26]	2013	China	endometrial cancer	1024	1918	Case:54.8±8.5 Control:50.7±9.5	PB	the Tagger program	0.52
Zenz [27]	2009	Germany	chronic lymphocytic leukemia	271	120	NR	PB	slow-down PCR using Eppendorf Taq PCR Mastermix	0.52

HB: hospital based; PB: population based; PCR-RFLP: polymerase chain reaction-restriction fragment length polymorphism; NR: no report; HWE, Hardy-Weinberg equilibrium

Table S2. Baseline characteristics of studies evaluating cancer prognosis included in the meta-analysis.

Study	Year	Country	Tumor Type	Sample/male	Age(year)	Detection method	HR estimation	median follow-up date (months)
Hirata [28]	2008	Japan	renal cancer	216/149	mean value: 62.2±12	PCR-RFLP	R	NR
Moon [29]	2010	Korea	acute myeloid leukemia	99/44	median: 48	PCR in a LightCycler-System	E	13
Zenz [27]	2009	Germany	chronic lymphocytic leukemia	271	median: 56	slow-down PCR using Eppendorf Taq PCR Mastermix	R	78
Xu [23]	2013	China	Lung cancer	1017	NR	TaqMan method using the ABI 7900HT Sequence Detection System	R	>48
Knoefel [30]	2011	Germany	Lung Cancer	188/141	NR	PCR in a LightCycler-System	R	NR
Bachmann [31]	2011	Germany	prostate cancer	142/142	mean value: 65.7±5.6	slow-down PCR analyzed using the PSQ96 SNP software	R	51
Bachmann [32]	2007	Germany	breast cancer	274/0	NR	slow-down PCR analyzed using the PSQ96 SNP software	R	80
Kaderi [33]	2008	Sweden	chronic lymphocytic leukemia	268	NR	PCR and SBE-FP	E	95
Künkele [34]	2013	Germany	acute lymphoblastic leukemia	182/111	mean value: 6.5±4.6	slow-down PCR analyzed using the PSQ96 SNP software	E	62

Lehnerdt [35]	2009	Germany	squamous cell carcinoma	133/105	mean value: 59.4±10.7	slow-down PCR analyzed using the PSQ96 SNP software	E	60
Nückle [36]	2007	Germany	chronic lymphocytic leukemia	123/86	mean value： 60	slow-down PCR analyzed using the PSQ96 SNP software	R	NR
Masago [37]	2013	Japan	lung cancer	NR	NR	PCR and the 5'-nuclease assay	R	NR
Hindy [38]	2011	Germany	glioblastoma	160/109	mean value： 58±13.7	slow-down PCR analyzed using the PSQ96 SNP software	R	24
Heubner [39]	2009	Germany	ovarian cancer	110/0	median: 58	slow-down PCR analyzed using the PSQ96 SNP software	R	25

PCR-RFLP: polymerase chain reaction-restriction fragment length polymorphism; NR: no report; E: estimated; R: reported; SBE-FP: single-base primer extension with detection by fluorescence polarization.

Table S3. A summary of odds ratios (ORs) for the overall and subgroup analyses of *Bcl-2* -938 C>A polymorphism and cancer susceptibility.

Subgroups	Dominant model (ORs)	95%CI	P value	Recessive Model (ORs)	95%CI	P value	Allelic model(ORs)	95%CI	P value
Overall	1.12	1.00-1.25	0.04	1.38	1.11-1.71	0.004	1.15	1.04-1.28	0.007
Asians	1.19	1.08-1.31	0.0005	1.83	1.28-2.62	0.0009	1.28	1.12-1.47	0.0003
Caucasians	0.96	0.79-1.16	0.65	1.04	0.89-1.21	0.82	1.00	0.89-1.12	0.97
HB	1.14	0.92-1.42	0.22	1.53	1.08-2.17	0.02	1.20	1.00-1.44	0.05
PB	1.11	1.00-1.24	0.05	1.23	0.92-1.64	0.16	1.11	1.00-1.23	0.06
Subgroups	Homozygote comparison (ORs)	95%CI	P value	Heterozygote comparison (ORs)	95%CI	P value			
Overall	1.44	1.11-1.87	0.02	1.05	0.97-1.14	0.22			
Asians	1.96	1.35-2.85	0.006	1.11	1.00-1.23	0.04			
Caucasians	0.98	0.75-1.29	0.91	0.94	0.80-1.11	0.48			
HB	1.59	1.02-2.50	0.04	1.03	0.88-1.22	0.71			
PB	1.29	0.95-1.76	0.10	1.08	0.97-1.21	0.17			

ORs: odds ratios; CI: confidence interval; PB: population based; HB: hospital based.

Table s4. The influence of individual study on the pooled estimate (OR) in allelic model

Study omitted	Year	OR	95%CI	P-value	Heterogeneity	
					I^2	P value
None		1.15	1.04-1.28	0.007	68	<0.001
Chen [21]	2007	1.18	1.05-1.32	0.006	68	<0.001
Dorjgochoo[26]	2013	1.17	1.03-1.32	0.01	71	<0.001
Fingas [24]	2010	1.17	1.05-1.30	0.004	69	<0.001
Hyndman[16]	2011	1.19	1.07-1.32	0.0009	61	0.004
Li [17]	2014	1.14	1.02-1.27	0.02	68	<0.001
Liu [22]	2012	1.14	1.02-1.26	0.02	68	<0.001
Meyer [25]	2013	1.15	1.03-1.29	0.01	70	<0.001
Wang [19]	2012	1.13	1.02-1.25	0.02	68	<0.001
Wang [20]	2014	1.11	1.01-1.22	0.03	57	0.01
Xu [23]	2013	1.17	1.04-1.32	0.01	70	<0.001
Zenz [27]	2009	1.17	1.04-1.30	0.006	70	<0.001
Zhang [18]	2011	1.14	1.03-1.27	0.01	69	<0.001

OR, odds ratio; CI, confidence interval. P<0.05 denoted statistical significance.

Table s5. The influence of individual study on the pooled estimate (OR) in dominant model

Study omitted	Year	OR	95%CI	P-value	Heterogeneity	
					I^2	P value
None		1.12	1.00-1.25	0.04	38	0.09
Chen [21]	2007	1.14	1.01-1.29	0.03	39	0.09
Dorjgochoo[26]	2013	1.12	0.98-1.28	0.09	44	0.06
Fingas [24]	2010	1.13	1.01-1.26	0.04	42	0.07
Hyndman[16]	2011	1.15	1.06-1.25	0.001	0	0.54
Li [17]	2014	1.11	0.99-1.24	0.09	41	0.08
Liu [22]	2012	1.11	0.99-1.25	0.08	42	0.07
Meyer [25]	2013	1.11	0.99-1.26	0.07	43	0.08
Wang [19]	2012	1.10	0.99-1.23	0.08	35	0.12
Wang [20]	2014	1.09	0.98-1.22	0.12	31	0.15
Xu [23]	2013	1.13	1.00-1.29	0.06	43	0.06
Zenz [27]	2009	1.13	1.00-1.27	0.04	43	0.06
Zhang [18]	2011	1.12	0.99-1.25	0.07	43	0.06

OR, odds ratio; CI, confidence interval. $P<0.05$ denoted statistical significance.

Table s6. The influence of individual study on the pooled estimate (OR) in recessive model

Study omitted	Year	OR	95%CI	P-value	Heterogeneity	
					I^2	P value
None		1.38	1.11-1.71	0.004	74	<0.001
Chen [21]	2007	1.44	1.13-1.85	0.004	75	<0.001
Dorjgochoo[26]	2013	1.43	1.11-1.85	0.005	76	<0.001
Fingas [24]	2010	1.42	1.14-1.76	0.001	75	<0.001
Hyndman[16]	2011	1.45	1.14-1.84	0.002	74	<0.001
Li [17]	2014	1.33	1.07-1.66	0.01	74	<0.001
Liu [22]	2012	1.31	1.06-1.63	0.01	73	<0.001
Meyer [25]	2013	1.36	1.06-1.74	0.007	76	<0.001
Wang [19]	2012	1.33	1.07-1.65	0.01	74	<0.001
Wang [20]	2014	1.24	1.04-1.49	0.02	60	<0.001
Xu [23]	2013	1.43	1.12-1.83	0.005	76	<0.001
Zenz [27]	2009	1.42	1.13-1.79	0.003	76	<0.001
Zhang [18]	2011	1.34	1.08-1.67	0.008	75	<0.001

OR, odds ratio; CI, confidence interval. P<0.05 denoted statistical significance.

Table s7. The influence of individual study on the pooled estimate (OR) in homozygote comparison

Study omitted	Year	OR	95%CI	P-value	Heterogeneity	
					I^2	P value
None		1.44	1.11-1.87	0.006	77	<0.001
Chen [21]	2007	1.51	1.12-2.03	0.006	78	<0.001
Dorjgochoo[26]	2013	1.49	1.09-2.03	0.01	79	<0.001
Fingas [24]	2010	1.45	1.09-1.93	0.01	81	<0.001
Hyndman[16]	2011	1.55	1.19-2.02	0.001	74	<0.001
Li [17]	2014	1.39	1.06-1.82	0.02	77	<0.001
Liu [22]	2012	1.37	1.05-1.78	0.02	76	<0.001
Meyer [25]	2013	1.45	1.08-1.93	0.01	79	<0.001
Wang [19]	2012	1.37	1.05-1.78	0.02	77	<0.001
Wang [20]	2014	1.30	1.03-1.64	0.03	67	<0.001
Xu [23]	2013	1.50	1.11-2.02	0.009	79	<0.001
Zenz [27]	2009	1.48	1.12-1.96	0.005	79	<0.001
Zhang [18]	2011	1.39	1.07-1.82	0.01	78	<0.001

OR, odds ratio; CI, confidence interval. P<0.05 denoted statistical significance.

Table s8. The influence of individual study on the pooled estimate (OR) in heterozygote comparison

Study omitted	Year	OR	95%CI	P-value	Heterogeneity	
					I^2	P value
None		1.05	0.97-1.14	0.22	0	0.59
Chen [21]	2007	1.07	0.97-1.17	0.16	0	0.55
Dorjgochoo[26]	2013	1.03	0.94-1.14	0.52	0	0.56
Fingas [24]	2010	1.05	0.97-1.15	0.22	0	0.50
Hyndman[16]	2011	1.09	1.00-1.19	0.06	0	0.99
Li [17]	2014	1.05	0.96-1.14	0.28	0	0.52
Liu [22]	2012	1.05	0.97-1.14	0.25	0	0.50
Meyer [25]	2013	1.05	0.96-1.14	0.31	0	0.52
Wang [19]	2012	1.05	0.96-1.14	0.31	0	0.81
Wang [20]	2014	1.04	0.96-1.14	0.33	0	0.54
Xu [23]	2013	1.05	0.96-1.16	0.26	0	0.50
Zenz [27]	2009	1.05	0.97-1.15	0.22	0	0.50
Zhang [18]	2011	1.05	0.97-1.14	0.24	0	0.50

OR, odds ratio; CI, confidence interval. $P<0.05$ denoted statistical significance.

Table S9. A summary of P values for Begg’s funnel plot and Egger’s test in five genetic models.

	Begg's funnel plot	Egger's test
Dominant model	1	0.6091
Recessive model	0.1702	0.06691
Homozygote comparison	0.337	0.1708
Heterozygote comparison	0.4106	0.9256
Allelic model	0.8229	0.2497

Table s10. The influence of individual study on the pooled estimate (HR) in AA versus CA

Study omitted	Year	HR	95%CI	P-value	Heterogeneity	
					I^2	P value
None		0.99	0.77-1.27	0.93	37	0.15
Bachmann [32]	2007	1.00	0.73-1.38	1.00	46	0.10
Kaderi [33]	2008	1.06	0.75-1.51	0.74	44	0.11
Knoefel [30]	2011	1.04	0.75-1.44	0.81	46	0.10
Lehnerdt [35]	2009	1.02	0.76-1.36	0.91	47	0.09
Moon [29]	2010	0.94	0.74-1.20	0.62	32	0.20
Zenz (Essen) [27]	2009	0.92	0.77-1.10	0.34	0	0.64
Zenz (Ulm) [27]	2009	1.04	0.79-1.38	0.77	42	0.13

HR, hazard ratio; CI, confidence interval. $P<0.05$ denoted statistical significance.

Table s11. The influence of individual study on the pooled estimate (HR) in AA versus CC

Study omitted	Year	HR	95%CI	P-value	Heterogeneity	
					I^2	P value
None		0.92	0.65-1.30	0.63	65	0.002
Bachmann [32]	2007	0.94	0.63-1.40	0.76	69	0.001
Bachmann [31]	2011	0.87	0.63-1.19	0.38	62	0.007
Hindy [38]	2011	0.82	0.59-1.15	0.26	57	0.02
Kaderi [33]	2008	0.96	0.62-1.49	0.87	69	0.001
Knoefel [30]	2011	1.01	0.72-1.41	0.96	58	0.02
Lehnerdt [35]	2009	0.97	0.67-1.41	0.89	67	0.002
Moon [29]	2010	0.89	0.62-1.28	0.52	68	0.002
Xu [23]	2013	0.93	0.62-1.40	0.72	69	0.001
Zenz (Essen) [27]	2009	0.87	0.63-1.21	0.42	64	0.004
Zenz (Ulm) [27]	2009	0.97	0.67-1.40	0.88	68	0.002

HR, hazard ratio; CI, confidence interval. $P<0.05$ denoted statistical significance.

Table s12. The influence of individual study on the pooled estimate (HR) in CA versus CC

Study omitted	Year	HR	95%CI	P-value	Heterogeneity	
					I^2	P value
None		0.94	0.80-1.11	0.48	14	0.32
Bachmann [31]	2011	0.95	0.84-1.07	0.39	0	0.42
Hindy [38]	2011	0.91	0.76-1.10	0.35	20	0.28
Kaderi [33]	2008	0.91	0.68-1.23	0.55	28	0.23
Lehnerdt [35]	2009	0.98	0.86-1.10	0.68	0	0.59
Moon [29]	2010	0.94	0.76-1.15	0.53	31	0.21
Xu [23]	2013	0.95	0.74-1.22	0.69	29	0.23

HR, hazard ratio; CI, confidence interval. P<0.05 denoted statistical significance.

Table s13. The influence of individual study on the pooled HRs in CC versus CA+AA

Study omitted	Year	HR	95%CI	P-value	Heterogeneity	
					I^2	P value
None		1.21	0.69-2.13	0.50	78	0.004
Hirata [28]	2008	1.04	0.58-1.87	0.90	76	0.01
Künkele [34]	2013	1.41	0.69-2.86	0.34	85	0.001
Lehnerdt [35]	2009	1.03	0.57-1.86	0.93	67	0.05
Masago [37]	2013	1.49	0.79-2.78	0.22	57	0.1

HR, hazard ratio; CI, confidence interval. P<0.05 denoted statistical significance.

Table s14. The influence of individual study on the pooled HRs in AA versus CC+CA

Study omitted	Year	HR	95%CI	P-value	Heterogeneity	
					I^2	P value
None		0.99	0.48-2.04	0.97	83	<0.001
Heubner [39]	2009	1.31	0.65-2.64	0.45	81	0.001
Knoefel [30]	2011	1.08	0.37-3.11	0.89	87	<0.001
Nückel [36]	2007	0.82	0.36-1.86	0.63	81	0.001
Zenz (Essen) [27]	2009	0.75	0.38-1.48	0.40	81	0.001
Zenz (Ulm) [27]	2009	1.11	0.43-2.86	0.83	87	0.001

HR, hazard ratio; CI, confidence interval. P<0.05 denoted statistical significance.

Table S15 Begg's funnel plot and Egger's test of publication bias on the relationships between Bcl-2 -938 C>A polymorphism and cancer prognosis in five genetic models.

	Begg's funnel plot	Egger's test
AA vs CA	0.095	0.121
AA vs CC	0.419	0.308
CA vs CC	0.707	0.825
CC vs AA+CA	1	0.321
AA vs CA+CC	1	0.836

Supplementary Figure S1. Forest plot of *Bcl-2* -938-C/A polymorphism and cancer risk in heterozygote comparison.

Heterozygote comparison (CA vs CC)

	Experimental		Control			Odds Ratio
**Study or Subgroup**	**Events**	**Total**	**Events**	**Total**	**Weight**	**M-H, Random, 95% CI**
Chen 2007	382	608	446	703	13.7%	0.97 [0.78, 1.22]
Dorjgochoo 2013	502	867	908	1647	25.2%	1.12 [0.95, 1.32]
Fingas 2010	21	35	18	30	0.7%	1.00 [0.37, 2.71]
Hyndman 2014	345	514	282	382	8.1%	0.72 [0.54, 0.97]
Li 2014	103	212	105	233	5.0%	1.15 [0.79, 1.67]
Liu 2012	96	171	112	208	4.2%	1.10 [0.73, 1.65]
Meyer 2013	259	405	238	390	8.4%	1.13 [0.85, 1.51]
Wang 2012	58	96	102	194	2.8%	1.38 [0.84, 2.26]
Wang 2014	198	345	223	414	8.4%	1.15 [0.87, 1.54]
Xu 2013	483	861	479	872	19.4%	1.05 [0.87, 1.27]
Zenz 2009	139	186	63	84	2.0%	0.99 [0.54, 1.79]
Zhang 2011	53	95	53	99	2.2%	1.10 [0.62, 1.93]
**Total (95% CI)**		**4395**		**5256**	**100.0%**	**1.05 [0.97, 1.14]**
Total events	2639		3029			

Heterogeneity: $Tau^2 = 0.00$; $Chi^2 = 9.36$, df = 11 (P = 0.59); $I^2 = 0\%$

Test for overall effect: Z = 1.22 (P = 0.22)

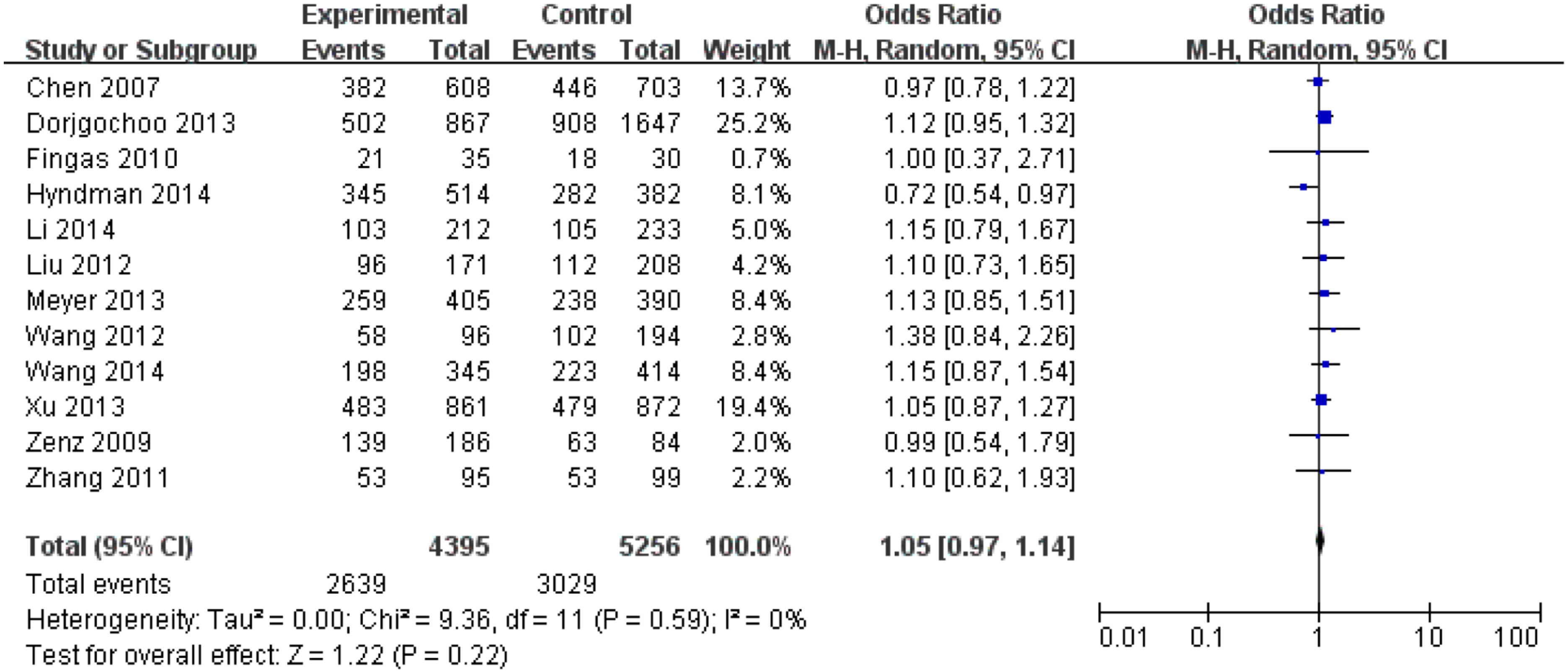

Supplementary Figure S2. Begg's funnel plot of publication bias on the relationships between *Bcl-2* -938 C>A polymorphism and susceptibility to cancer in five genetic models (A) Begg's funnel plot of publication bias on the relationships between *Bcl-2* -938 C>A polymorphism and susceptibility to cancer in dominant model; (B) Begg's funnel plot of publication bias on the relationships between *Bcl-2* -938 C>A polymorphism and susceptibility to cancer in recessive model; (C) Begg's funnel plot of publication bias on the relationships between *Bcl-2* -938 C>A polymorphism and susceptibility to cancer in homozygote comparison; (D) Begg's funnel plot of publication bias on the relationships between *Bcl-2* -938 C>A polymorphism and susceptibility to cancer in heterozygote comparison; (E) Begg's funnel plot of publication bias on the relationships between *Bcl-2* -938 C>A polymorphism and susceptibility to cancer in allelic model.

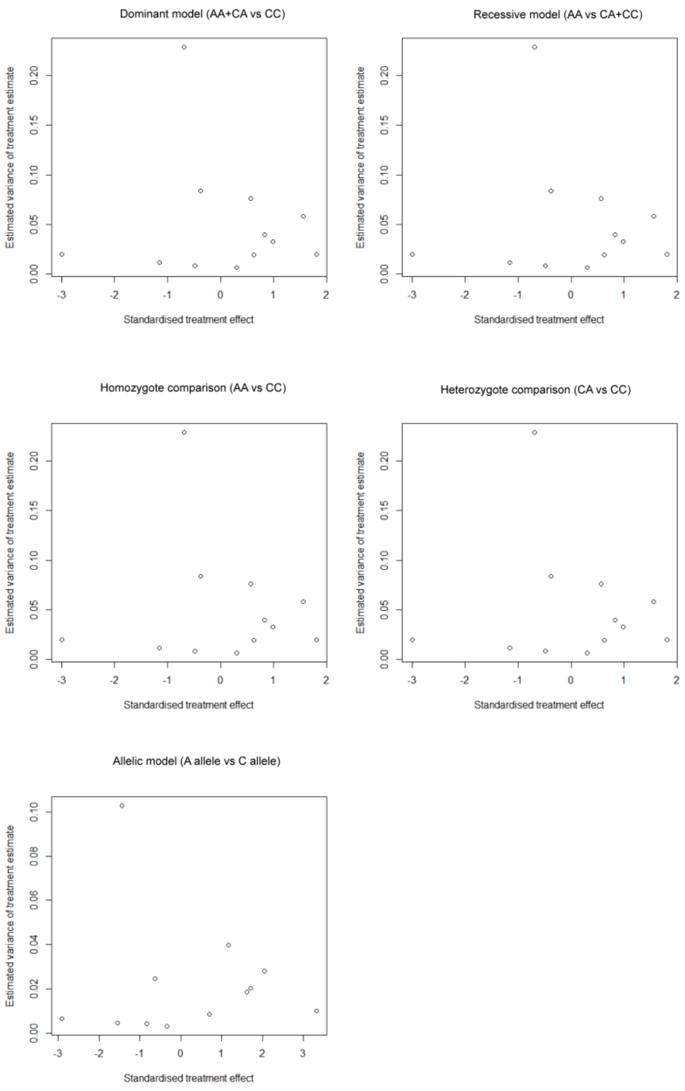
Dominant model (AA+CA vs CC)
Recessive model (AA vs CA+CC)
Homozygote comparison (AA vs CC)
Heterozygote comparison (CA vs CC)
Allelic model (A allele vs C allele)
Estimated variance of treatment estimate
Standardised treatment effect
0.00
0.05
0.10
0.15
0.20
0.02
0.04
0.06
0.08
-3
-2
-1
0
1
2
3

Supplementary Figure S3. Egger's test of publication bias on the relationships between *Bcl-2* -938 C>A polymorphism and susceptibility to cancer in five genetic models (A) Egger's test of publication bias on the relationships between *Bcl-2* -938 C>A polymorphism and susceptibility to cancer in dominant model; (B) Egger's test of publication bias on the relationships between *Bcl-2* -938 C>A polymorphism and susceptibility to cancer in recessive model; (C) Egger's test of publication bias on the relationships between *Bcl-2* -938 C>A polymorphism and susceptibility to cancer in homozygote comparison; (D) Egger's test of publication bias on the relationships between *Bcl-2* -938 C>A polymorphism and susceptibility to cancer in heterozygote comparison; (E) Egger's test of publication bias on the relationships between *Bcl-2* -938 C>A polymorphism and susceptibility to cancer in allelic model.

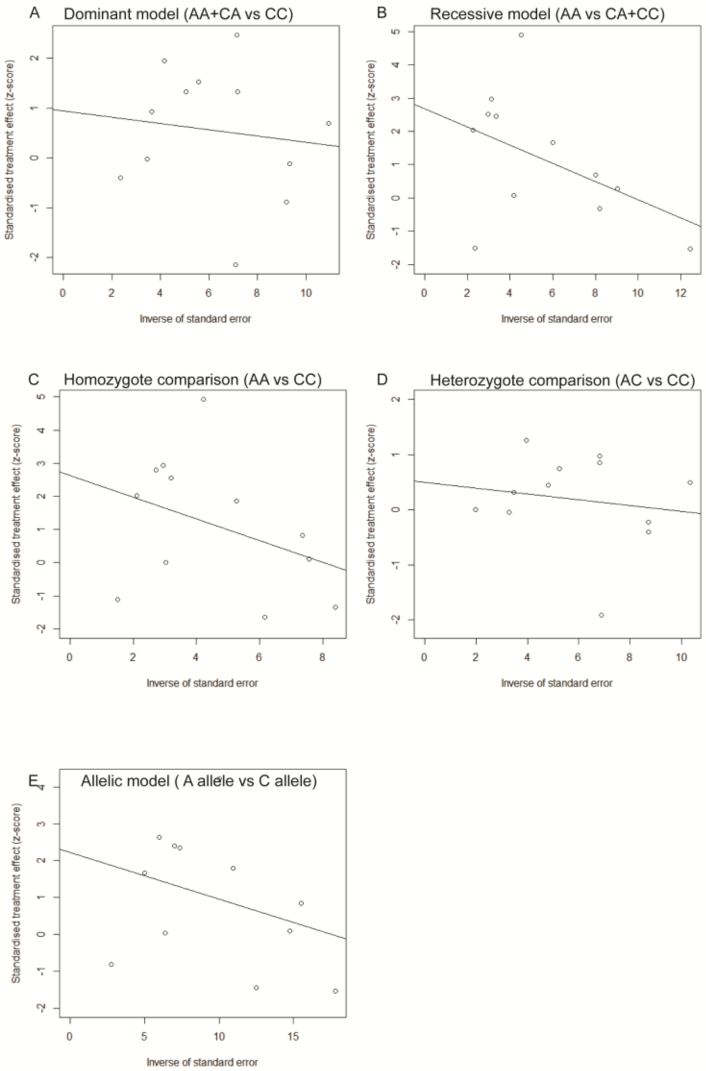
A
Dominant model (AA+CA vs CC)
Standardised treatment effect (z-score)
Inverse of standard error
B
Recessive model (AA vs CA+CC)
Standardised treatment effect (z-score)
Inverse of standard error
C
Homozygote comparison (AA vs CC)
Standardised treatment effect (z-score)
Inverse of standard error
D
Heterozygote comparison (AC vs CC)
Standardised treatment effect (z-score)
Inverse of standard error
E
Allelic model (A allele vs C allele)
Standardised treatment effect (z-score)
Inverse of standard error

Supplementary Figure S4. **Forest plot of all cancer samples treated as a cancer group against the control group to evaluate the significance of the odds ratios in dominant model.**

Study or Subgroup	Experimental Events	Experimental Total	Control Events	Control Total	Weight	Odds Ratio M-H, Random, 95% CI
overall group	3759	5515	4063	6290	100.0%	1.17 [1.09, 1.27]
**Total (95% CI)**		**5515**		**6290**	**100.0%**	**1.17 [1.09, 1.27]**
Total events	3759		4063			
Heterogeneity: Not applicable						
Test for overall effect: Z = 4.09 (P < 0.0001)						

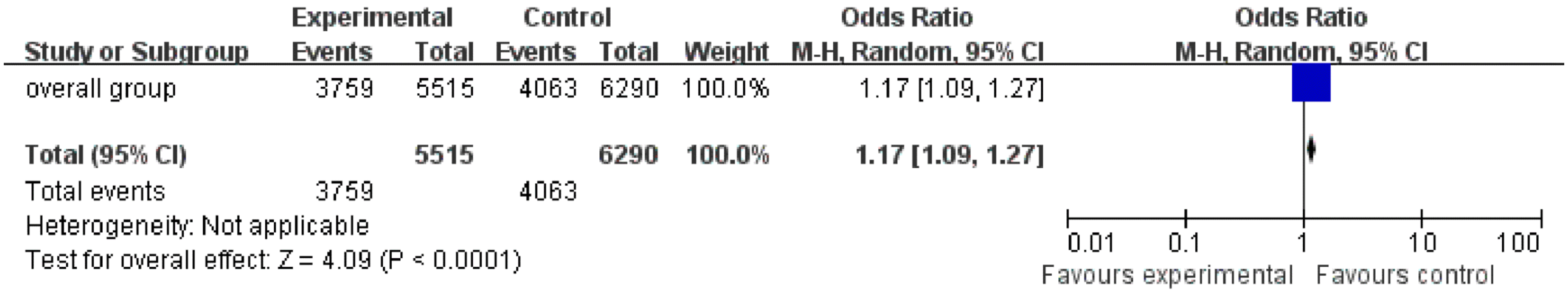

Supplementary Figure S5. Forest plot of all cancer samples treated as a cancer group against the control group to evaluate the significance of the odds ratios in recessive model.

Study or Subgroup	Experimental Events	Experimental Total	Control Events	Control Total	Weight	Odds Ratio M-H, Random, 95% CI
overall group	1120	5515	1034	6290	100.0%	1.30 [1.18, 1.42]
**Total (95% CI)**		**5515**		**6290**	**100.0%**	**1.30 [1.18, 1.42]**
Total events	1120		1034			
Heterogeneity: Not applicable						
Test for overall effect: Z = 5.42 (P < 0.00001)						

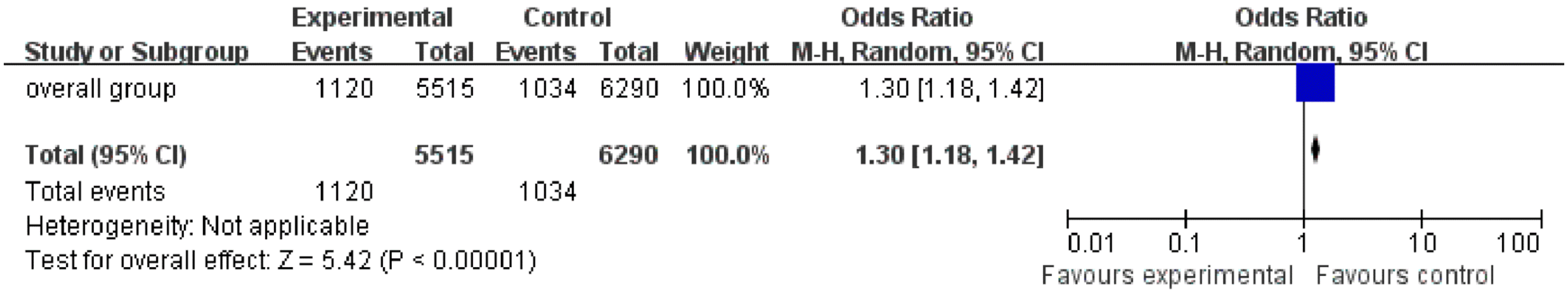

Supplementary Figure S6. Forest plot of all cancer samples treated as a cancer group against the control group to evaluate the significance of the odds ratios in homozygote comparison.

	Experimental		Control			Odds Ratio	Odds Ratio
**Study or Subgroup**	**Events**	**Total**	**Events**	**Total**	**Weight**	**M-H, Random, 95% CI**	**M-H, Random, 95% CI**
overall group	1120	2876	1034	3261	100.0%	1.37 [1.24, 1.53]	
**Total (95% CI)**		**2876**		**3261**	**100.0%**	**1.37 [1.24, 1.53]**	
Total events	1120		1034				
Heterogeneity: Not applicable							
Test for overall effect: Z = 5.92 (P < 0.00001)							

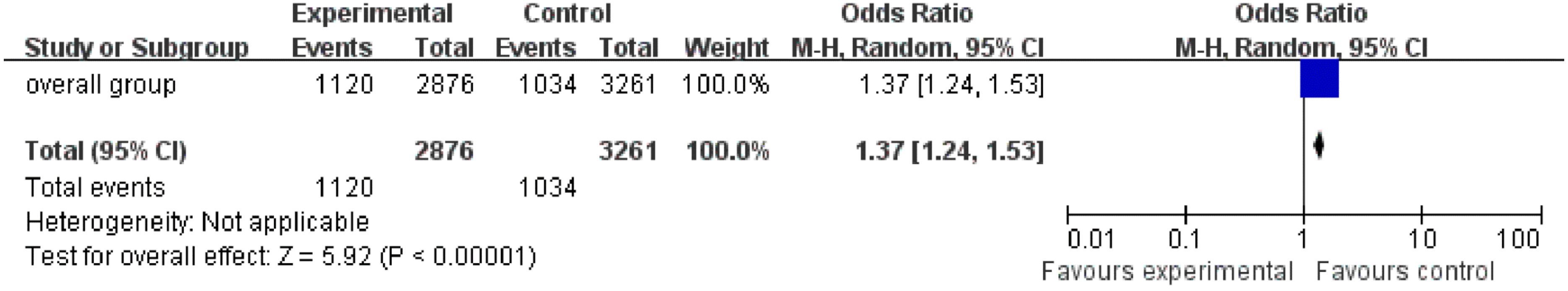

Supplementary Figure S7. Forest plot of all cancer samples treated as a cancer group against the control group to evaluate the significance of the odds ratios in heterozygote comparison.

	Experimental		Control			Odds Ratio	Odds Ratio
**Study or Subgroup**	**Events**	**Total**	**Events**	**Total**	**Weight**	**M-H, Random, 95% CI**	**M-H, Random, 95% CI**
overall group	2639	4395	3029	5256	100.0%	1.10 [1.02, 1.20]	
**Total (95% CI)**		**4395**		**5256**	**100.0%**	**1.10 [1.02, 1.20]**	
Total events	2639		3029				

Heterogeneity: Not applicable

Test for overall effect: Z = 2.40 (P = 0.02)

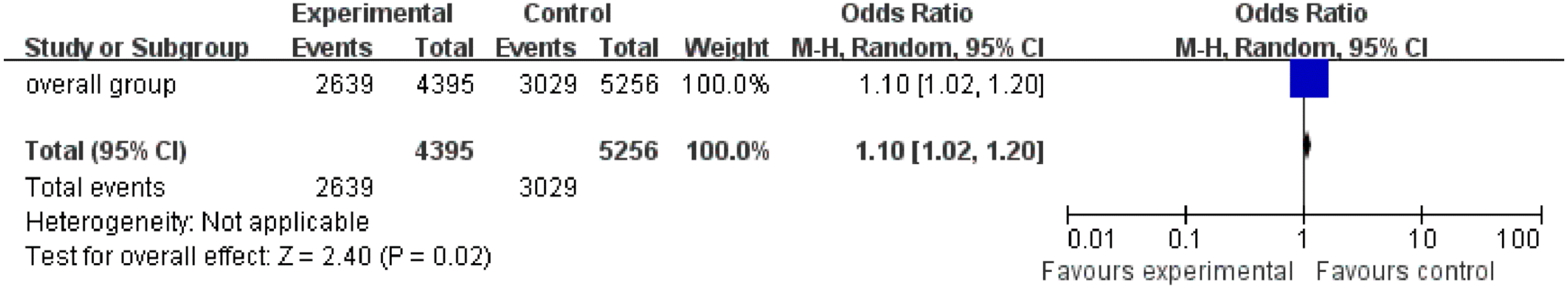

Supplementary Figure S8. Forest plot of all cancer samples treated as a cancer group against the control group to evaluate the significance of the odds ratios in allelic model.

	Experimental		Control			Odds Ratio
**Study or Subgroup**	**Events**	**Total**	**Events**	**Total**	**Weight**	**M-H, Random, 95% CI**
overall group	4879	11030	5097	12580	100.0%	1.16 [1.11, 1.23]
**Total (95% CI)**		**11030**		**12580**	**100.0%**	**1.16 [1.11, 1.23]**
Total events	4879		5097			
Heterogeneity: Not applicable						
Test for overall effect: Z = 5.77 (P < 0.00001)						

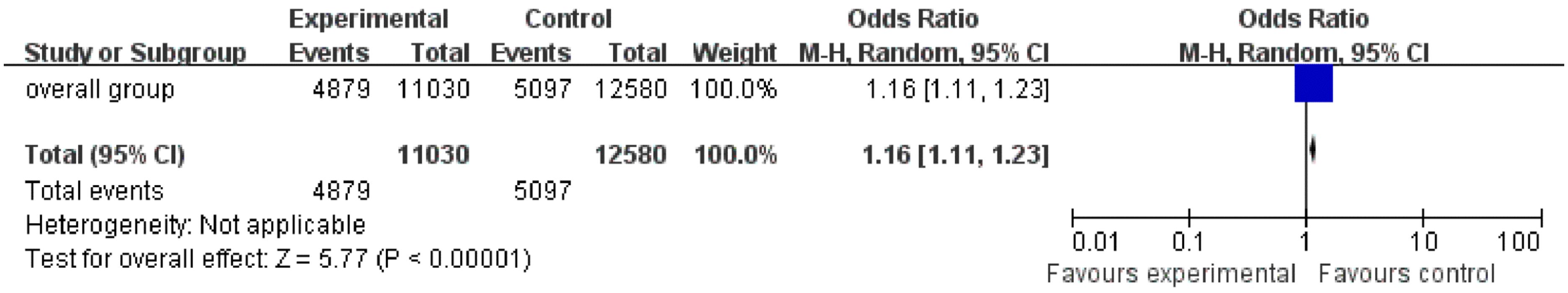

Supplementary Checklist S1. Preferred Reporting Items for Systematic Reviews and Meta-Analyses (PRISMA) Checklist.

Section/topic	#	Checklist item	Reported on page #
**TITLE**			
Title	1	Identify the report as a systematic review, meta-analysis, or both.	**Title**
**ABSTRACT**			
Structured summary	2	Provide a structured summary including, as applicable: background; objectives; data sources; study eligibility criteria, participants, and interventions; study appraisal and synthesis methods; results; limitations; conclusions and implications of key findings; systematic review registration number.	**Abstract**
**INTRODUCTION**			
Rationale	3	Describe the rationale for the review in the context of what is already known.	**Introduction**
Objectives	4	Provide an explicit statement of questions being addressed with reference to participants, interventions, comparisons, outcomes, and study design (PICOS).	**Introduction**
**METHODS**			
Protocol and registration	5	Indicate if a review protocol exists, if and where it can be accessed (e.g., Web address), and, if available, provide registration information including registration number.	**NA**
Eligibility criteria	6	Specify study characteristics (e.g., PICOS, length of follow-up) and repo rt characteristics (e.g., years considered, language, publication status) used as criteria for eligibility, giving rationale.	**Literature search and**

			**Selection criteria**
Information sources	7	Describe all information sources (e.g., databases with dates of coverage, contact with study authors to identify additional studies) in the search and date last searched.	**Literature search and Selection criteria**
Search	8	Present full electronic search strategy for at least one database, including any limits used, such that it could be repeated.	**Literature search and Selection criteria**
Study selection	9	State the process for selecting studies (i.e., screening, eligibility, included in systematic review, and, if applicable, included in the meta-analysis).	**Data abstraction**
Data collection process	10	Describe method of data extraction from reports (e.g., piloted forms, independently, in duplicate) and any processes for obtaining and confirming data from investigators.	**Data abstraction**
Data items	11	List and define all variables for which data were sought (e.g., PICOS, funding sources) and any assumptions and simplifications made.	**Statistical analysis**
Risk of bias in individual studies	12	Describe methods used for assessing risk of bias of individual studies (including specification of whether this was done at the study or outcome level), and how this information is to be used in any data synthesis.	**Data abstraction**
Summary measures	13	State the principal summary measures (e.g., risk ratio, difference in means).	**Statistical**

			**analysis**
Synthesis of results	14	Describe the methods of handling data and combining results of studies, if done, including measures of consistency (e.g., I^2) for each meta-analysis.	**Statistical analysis**

Section/topic	#	Checklist item	Reported on page #
Risk of bias across studies	15	Specify any assessment of risk of bias that may affect the cumulative evidence (e.g., publication bias, selective reporting within studies).	**Statistical analysis**
Additional analyses	16	Describe methods of additional analyses (e.g., sensitivity or subgroup analyses, meta-regression), if done, indicating which were pre-specified.	**Statistical analysis**
**RESULTS**			
Study selection	17	Give numbers of studies screened, assessed for eligibility, and included in the review, with reasons for exclusions at each stage, ideally with a flow diagram.	**Fig.1**
Study characteristics	18	For each study, present characteristics for which data were extracted (e.g., study size, PICOS, follow-up period) and provide the citations.	**Table S1 and Table S2**
Risk of bias within studies	19	Present data on risk of bias of each study and, if available, any outcome level assessment (see item 12).	**Fig. S1, Fig S2, Table 1 and Table s14**

Results of individual studies	20	For all outcomes considered (benefits or harms), present, for each study: (a) simple summary data for each intervention group (b) effect estimates and confidence intervals, ideally with a forest plot.	**Fig 2-5 and Fig S1,S2**
Synthesis of results	21	Present results of each meta-analysis done, including confidence intervals and measures of consistency.	**Table S3**
Risk of bias across studies	22	Present results of any assessment of risk of bias across studies (see Item 15).	**Fig. S1, Fig S2, Table 1 and Table S14**
Additional analysis	23	Give results of additional analyses, if done (e.g., sensitivity or subgroup analyses, meta-regression [see Item 16]).	**Table S3-S13**
**DISCUSSION**			
Summary of evidence	24	Summarize the main findings including the strength of evidence for each main outcome; consider their relevance to key groups (e.g., healthcare providers, users, and policy makers).	**Discussion**
Limitations	25	Discuss limitations at study and outcome level (e.g., risk of bias), and at review-level (e.g., incomplete retrieval of identified research, reporting bias).	**Discussion**
Conclusions	26	Provide a general interpretation of the results in the context of other evidence, and implications for future research.	**Discussion**
**FUNDING**			
Funding	27	Describe sources of funding for the systematic review and other support (e.g., supply of data); role of funders for the systematic review.	**Acknowledgment**

From: Moher D, Liberati A, Tetzlaff J, Altman DG, The PRISMA Group (2009). Preferred Reporting Items for Systematic Reviews and Meta-Analyses: The PRISMA Statement. PLoS Med 6(6): e1000097. doi:10.1371/journal.pmed1000097

For more information, visit: **www.prisma-statement.org**.
